# Management of acquired prostatic fistulas in adults

**DOI:** 10.1007/s11255-024-04092-8

**Published:** 2024-06-27

**Authors:** Radion Garaz, Cristian Mirvald, Bastian Amend, Cristian Surcel, Igor Tsaur

**Affiliations:** 1 Department of Urology, University Hospital Tübingen, Hoppe-Seyler-Straße 3, 72076 Tübingen, Germany; 2 Department of Urology, Fundeni Clinical Institute, Bucharest, Romania; 3 University of Medicine and Pharmacy, ‘Carol Davila’ Bucharest, Bucharest, Romania

**Keywords:** Complication, Prostatic fistula, Surgical flap, Colorectal cancer, Prostate cancer

## Abstract

**Purpose:**

Acquired prostatic fistula (PF) was defined as a connection between the prostatic urethra and the rectum, symphysis, peritoneum, or ending freely in the periprostatic area. This study aims to report our experience with PF presentation, diagnosis, and treatment.

**Methods:**

From January 2014 to February 2024, we retrospectively analyzed a prospectively maintained database from two urologic university hospitals to identify men with acquired PF. Diagnosis was based on post-intervention symptoms, including pneumaturia, fecaluria, rectal urine leakage, periprostatic inflammation or abscess, completed by radiological assessment using retrograde urethrogram, CT, or MRI. Standard cystoscopy and/or rectosigmoidoscopy assessed bladder and rectal integrity. Patients with post-prostatectomy fistulas were excluded.

**Results:**

Thirteen patients with a mean age of 66.54 ± 7.40 years were identified. The most commonly presenting symptoms were fecaluria/pneumaturia 54%, rectal urine leakage 31%, and recurrent urinary tract infection 31%. The mean time from the initial treatment to fistula development was 22.28 ± 20.53 months (0.1–59 months), and from diagnosis to repair was 3.5 ± 3 months (1–12 months). Cumulative closure rates (success rate) post-first and second attempts were 77% (10 patients) and 92% (12 patients), respectively; one patient declined definitive surgery, maintaining a persistent fistula after bladder drainage.

**Conclusion:**

Clinical suspicion and detailed diagnosis are essential for formulating a tailored treatment plan for prostatic fistulas, which are successfully manageable in many patients. Complex cases benefit from a multidisciplinary approach, with individualized therapy based on etiology, severity, and recurrence of PF, facilitating effective closure.

## Introduction

The spectrum of genitourinary fistulas (GUF) in adults includes urogynecologic (vesicovaginal, ureterovaginal, vesicouterine, and urerthrovaginal), uro-enteric (vesicoenteric, pyeloenteric, and rectourethral), and urovascular fistula [1]. The predominant causes of acquired GUFs in both women and men are iatrogenic, often resulting from surgical procedures, ablative therapies, and radiotherapy. Neoplasm, infection, or trauma can also lead to their development [1, 2]. Rectourethral fistulas (RUFs) represent an abnormal connection between the distal part of the rectum and the lower urinary tract, manifesting as fecaluria, pneumaturia, and urinary discharge through the anus [3, 4]. Unlike congenital RUFs, the literature does not categorize acquired RUFs into subtypes, such as prostatic fistulas (PFs) and bulbar fistulas [1–6].

Diagnosis of these fistulas relies on history, physical examination, and radiological imaging. Radiological assessments, including CT scans and MRI, along with diagnostic procedures, such as cystoscopy, rectoscopy, voiding cystourethrogram, and retrograde urethrography, are instrumental in delineating the fistula’s anatomy and identifying any associated urinary or colorectal pathology [2, 4, 6].

The scarcity of acquired PFs in adults, coupled with the limited research available, has hindered the development of a standardized management protocol. The management of RUFs is particularly challenging due to their rarity and complexity, which has led to the innovation of various surgical techniques for their closure. These techniques are broadly categorized into abdominal, abdominoperineal, or perineal. The cornerstone of successful treatment is the anatomical separation of urethra, bladder, and rectum, coupled with individual closure of each structure. This may be further enhanced by the strategic placement of a vascularized flap [1, 3, 4]. In the surgical repair of PFs, while the approach is similar to that used for classical RUFs, the presence of the prostate necessitates specific modifications to the surgical plan.

This study aims to provide a comprehensive overview of the epidemiology, clinical manifestation, diagnostic strategies, and therapeutic options for patients with acquired PFs who have not undergone prostatectomy.

## Materials and methods

During January 2014 and February 2024, a clinical database of two university urological centers (University Hospital Tübingen, Tübingen, Germany, and Fundeni Clinical Institute, Bucharest, Romania) was analyzed to identify men with PF. All fistulas were acquired, both iatrogenic and directly related to tumor growth in cases of advanced prostate cancer (PCa) or colorectal cancer (CRC). Iatrogenic PF developed as a consequence of the treatment of the primary pelvic tumor (radiation and/or surgery) or following treatment for benign pathology of the prostate. This study was approved by the Institutional Review Board University Hospital Tübingen 536/2021 BO2. The Institutional Review Board Fundeni Clinical Institute did not require approval for retrospective studies.

Electronic medical records were assessed. Data on the patient’s demographics, past medical histories, previous histories of operations, pelvic radiation, etiology of the fistula, location of the fistula, presenting symptoms, method of diagnosis, time to fistula development, time of fistula repair, number and method of procedures, type of urinary diversion, fecal diversion, and recurrences were recorded. The etiology of the PF was defined by evaluating the history of previous treatments or trauma.

**Fig. 1:**
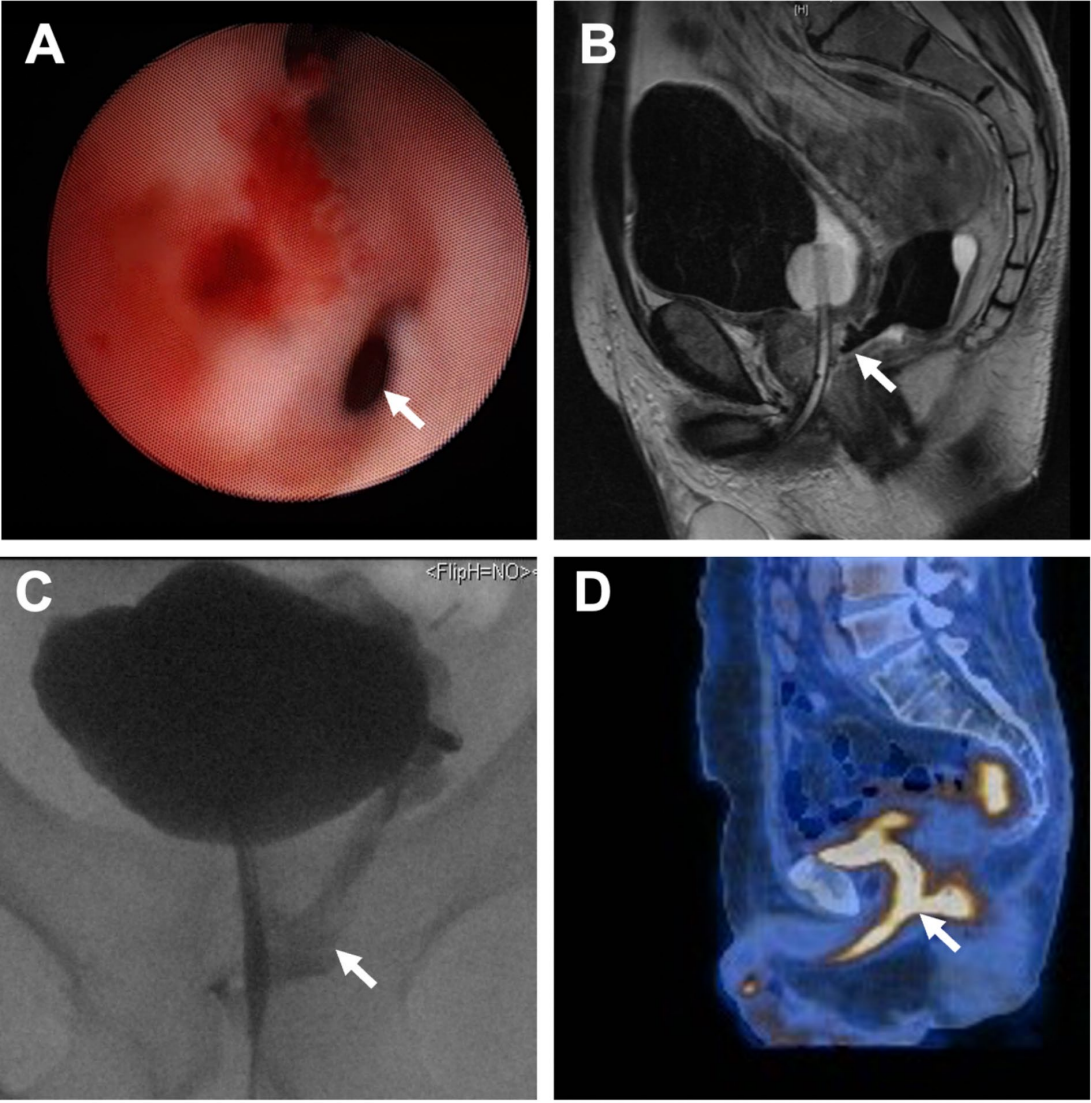
Methods of diagnosis of prostatic fistula. **A**. Cystoscopic view of the prostatic fistula after TURP (arrow); **B**. T2 MRI view of recto-prostatic fistula after trans-anal resection for gastrointestinal stromal tumor (arrow); **C**. Retrograde urethrogram view of the recto-prostatic fistula after colorectal anterior resection and radiotherapy for colorectal cancer; **D**. PET/CT view of a recto-prostatic fistula with associated urinoma in ischiorectal region following colorectal anterior resection and radiotherapy for colorectal cancer (arrow)

A PF was defined as an abnormal communication between the prostatic urethra and the rectum, symphysis, peritoneum, or ending freely in the periprostatic area, as shown in Fig. 1. PF was suspected based on a combination of preoperative symptoms, including pneumaturia, fecaluria, and rectal urine leakage, along with radiological findings from retrograde urethrogram and/or cross-sectional imaging via CT or MRI. Preoperative PET/CT scans were sometimes also conducted in cases with initial oncological diagnosis. Cystoscopy and/or rectosigmoidoscopy were routinely performed to assess the location of the fistula and proximity to the bladder neck, urethral sphincter, and rectum. Patients were included in the study if the fistula site directly contacted the prostate. Additionally, fistulas following prostatectomy for prostate cancer or orthotopic neobladder were excluded from the study.

Upon diagnosis, all patients underwent a bladder drainage, either suprapubic or transurethral catheter placement. When no spontaneous closure was achieved up to 4 months, a surgical repair of the fistula was attempted. Patients who failed conservative management were eligible to undergo definitive surgical repair. The surgical strategy was tailored to the anatomical complexity of the fistula, presence of sepsis, history of pelvic radiation, and residual urinary and fecal functionality. This was a similar preoperative strategy to the classical RUF [2, 4, 6]. The conservative techniques included suprapubic, transurethral catheter placement, or platelet-rich plasma (PRP) injection. Operative techniques included total prostatectomy (retropubic or robotic approach), transabdominal repair with omental flap interposition, en bloc fistula removal with cystoprostatectomy with ileal reservoir reconstruction, or transperineal repair with gracilis muscle flap interposition. Type and number of surgical procedures attempted to achieve successful fistula closure were recorded. Operations were performed by a urologic team and, in some complex cases, collaboratively with a colorectal surgeon or plastic surgeon.

A successful fistula closure is defined as the concurrent resolution of clinical symptoms and the absence of the fistula, as evidenced by radiological or endoscopic studies, which may also include the establishment of complete urinary and/or fecal diversion. Follow-up was obtained by reviewing the most recent outpatient clinical records on the hospital’s computer-based patient registry. Once healing was assessed, further follow-up was scheduled according to the initial diagnosis.

As there are no studies describing PFs, the literature review was conducted to identify studies focusing on RUFs. The primary outcome of the study was the presentation and diagnosis of PF. The secondary outcome focused on conservative treatment of the fistulas and their closure, based on the type of operation and the number of surgical attempts.

### Statistical analysis

Descriptive statistics were reported for continuous variables as median (range) and for categorical variables as n (%). All statistical analyses were performed using version 8 of GraphPad Prism (GraphPad Software, San Diego, California, USA, 2018).

## Results

Thirteen male patients with acquired PF were identified. In all cases, all of which had an iatrogenic origin. Four fistulas were related to PCa and seven to CRC. However, in one patient, the PF was a consequence of transurethral resection of the prostate (TURP) for a benign condition, and in another, it resulted from ulcerative colitis. Among these cases, eight patients were previously exposed to pelvic radiation, five after surgery for CRC, and three as a prior treatment for PCa. Patient characteristics, clinical presentation, diagnostic methods, and treatment modalities are detailed in Table 1.

**Table 1:** Detailed presentation of case series

No	Age	Initial diagnosis	Intervention	Radiotherapy	Time from intervention to symptoms (mo)	Symptoms	Diagnostic	Type of bladder drainage	Fecal diversion	Spontaneous closure (mo)	First Treatment	Second Treatment	Time from Fistulas to final Repair (mo)	Incontinence (After Fistula repair)	Follow-up (months)
Urinary pathology															
1	66	BPO	TURP	No	18	Pneumaturia	CU	TUC	No	4			4	No	6
2	69	PCa	IRE	No	3	Perineal PainDysuria	MRI	SPC	No	No	Robotic RP		1	No	4
3	70	PCa	HIFU	Yes	59	UTI	MRICUPET-CT	TUC	No	No	Salvage RP		1	Yes	30
4	73	PCa	CRR	Yes	25	UTIPerineal Pain	MRICU	TUC	No	No	RCP + SUD with IC		1		21
5	58	PCa	Radiation	Yes	49	Drainage of the urine per anus, Pneumaturia	COLCU PET-CT	SPC	Yes (temporary)	No	Retropubic RP + Omentum flap		4	No	6
Gastrointestinal pathology															
6	58	CRC	Colorectal anterior resection (Miles amputation)	Yes	6	Fecaluria Drainage of the urine per anus	CU PET-CT	SPC	Yes (definitive)	No	Unsuccessful RP	SUD Bricker’s	5		14
7	76	CRC	Colorectal anterior resection (Hartmann)	Yes	8.5	Fecaluria Drainage of the urine per anus	CU PET-CT	TUC	Yes (temporary)	No	Surgery-repair refused	Permanent SPC	0		
8	66	CRC	Colorectal anterior resection (Miles amputation)	Yes	0.1	Drainage of the urine per anus	CU CT	SPC	Yes (definitive)	No	PRP Injection	GMFI	12	No	4
9	67	CRC	Colorectal anterior resection	Yes	3	Fecaluria Dysuria	RUGMRI	TUC	Yes (temporary)	4	SPC		3	No	6
10	56	UC	Rectal resection with supraanal infralevatoric dissection	No	49	UTI	CU RUGMRI	TUC	Yes (definitive)	No	Reconstruction of prostatic urethra and GMFI		4	No	17
11	56	AC	Anus and Ileum-Pounch Holm Extirpation + right YV plasty	Yes	4	UTI Pneumaturia	CU RUGCT	SPC	Yes (definitive)	No	Retropubic RP		2	Yes	17
12	60	CRC	Colorectal anterior resection	Yes	29	Incontinence Fecaluria	CU RUGCT	TUC	Yes (temporary)	No	Retropubic RP		1	No	9
13	77	GIST	Transanal Resection	No	36	Fecaluria	CU RUGCOL MRI	TUC	Yes (temporary)	No	Retropubic RP + Omentum Flap		4	No	87

*BPO* benign prostatic obstruction, *TURP* transurethral resection of the prostate, *CU* cystoscopy, *TUC* transurethral catheter, *PCa* prostate cancer, *IRE* irreversible electroporation of prostate, *MRI* magnetic resonance imaging, *SPC* suprapubic urinary catheter, *RP* radical prostatectomy, *HIFU* high-intensity focused ultrasound, *UTI* urinary tract infection, *PET-CT* positron emission tomography-computed tomography, *CRR* cyberknife robotic radiosurgery, *RPC* radical cystoprostatectomy, *SUD* supravesical urinary diversion, *IC* ileum conduit, *COL* colonoscopy, *CRC* colorectal cancer, *PRP* platelet-rich plasma, *GMFI* gracilis muscle flap interposition, *RUG* retrograde urethrogram, *UC* ulcerative colitis, *AC* anal cancer, *CT* computed tomography, *GIST* gastrointestinal stromal tumor

The mean age at diagnosis was 66.54 ± 7.40 years (56–77 years). The most common presenting symptoms were fecaluria/pneumaturia in 54% of cases, urine leakage through the rectum/perineum in 31%, and recurrent urinary tract infections (UTIs) in 31%.

Initial management for all patients involved temporary bladder drainage through either a transurethral catheter (TUC) or a suprapubic catheter (SPC). The majority of patients (76.9%) underwent either definitive or temporary fecal diversion for their primary colorectal cancer. In contrast, 3 patients were scheduled for surgery immediately after diagnosis; therefore, no additional fecal diversion was performed. In two patients (15.5%), the fistula spontaneously closed after urinary diversion without further surgical interventions.

The decision to proceed with surgical intervention was made on a case-by-case basis, considering factors, such as size of the fistula, history of pelvic radiotherapy, and oncological status. The mean time between the initial intervention and fistula development was 22.28 ± 20.53 months (range 0.1–59 months), and the mean time from diagnosis and to final closure of the fistula, either conservatively or surgically was 3.5 ± 3 months (range 1–12 months).

Surgical approaches in our cohort included abdominal or combined abdominoperineal procedures. In 9 cases (69.2%), open or robotic total/radical prostatectomy was performed.

In 2 cases with complex fistulas following radiation therapy, cystoprostatectomy and supravesical urinary diversion (SUD) were required following the failure of 1st closure attempt.

**Fig. 2:**
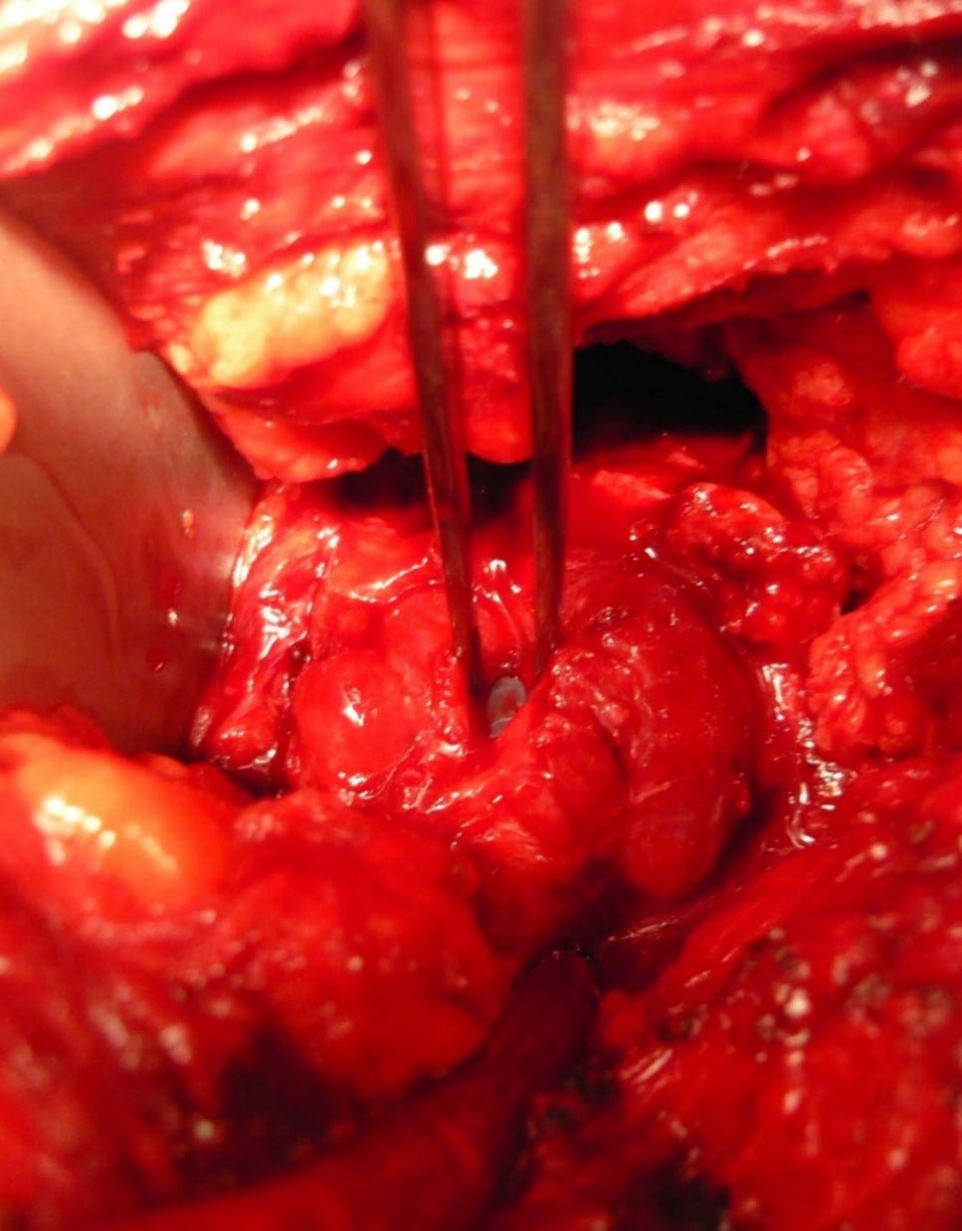
Intraoperative view of the prostatic fistula

The perineal approach was utilized in only 2 cases, primarily due to the challenges in accessing the fistula following significant anatomical changes from previous surgery (e.g., Miles amputation, lower anterior resection). Flap interposition was employed in four patients (31%), with omentum flaps used in two cases through an abdominal approach, Fig. 2, and gracilis muscle in two cases through a perineal approach.

Eight patients (61.5%) in our cohort achieved healing after the initial attempt, while 2 cases (15.4%) required a second intervention. The mean time between the first surgical closure of the fistula and the second repair was 7 ± 1.41 months.

Overall, closure of the fistula was achieved in 12 out of 13 patients (92.3%). Persistence of the fistula was observed in one patient who declined further definitive surgery after receiving only bladder drainage.

## Discussion

To the best of our knowledge, we present the first and most extensive cohort focused on acquired PFs. Managing PFs poses technical challenges due to anatomical complexities preceding complicating factors, such as tissue damage from radiation or ablation, infection, and concomitant neoplasia. Nevertheless, we described diagnostic possibilities and therapeutic strategies for addressing this complication following pelvic surgery for either prostate or colorectal diseases.

Uroenteric fistula can be caused by radiotherapy alone. The reported studies did not differentiate PF from RUF. Any type of radiation is currently related to around 50% of RUFs [1, 4]. The presumed pathophysiology associated with local irradiation across the rectal wall is microvascular injury due to local tissue hypoxia that causes mucosal damage and ischemia, leading to ulcers, perforations, and fistula formation. The estimated rate of RUF after external beam radiotherapy (EBRT) is about 1% and about 3% in the case of brachytherapy (BT) [4]. In a cohort of 51 patients treated for PCa at the Mayo Clinic, RUF incidence after EBRT, BT, and combined EBRT + BT was 30%, 30%, and 40%, respectively [7]. Following high-intensity focused ultrasound (HIFU) therapy, the rates of RUF are reported to be less than 3% after a single HIFU session for localized PCa [8]. Patients undergoing salvage HIFU therapy and repeated HIFU sessions have the highest overall risk for RUF, exceeding 5% [8]. Of the 471 patients treated with Irreversible Electroporation of the Prostate (IRE) and minimum follow-up for 4 months, Guenther et al. [9] reported a prostatic fistula in one patient (0.2%), which closed spontaneously after a few weeks.

In one case of our series, during colorectal resection for CRC, there was a prostatic injury, which developed a fistula, and the surgical repair was performed in 4 days. In the context of radical prostatectomy, rectal injuries are the main factor in the development of RUFs, with a correlation of up to 54% [4]. Overall, RUFs represent a relatively rare complication after prostatectomy, occurring in around 0.53% of cases [2, 4, 10].

In our study, the most relevant symptoms were fecaluria at 39%, rectal urine leakage at 31%, recurrent UTIs at 31%, pneumaturia at 23%, perineal pain at 15%, and dysuria at 15%. Compared to RUF characteristics, signs, and symptoms, fecaluria is present in about 43–65%, pneumaturia 67–85%, the leakage of urine through the rectum during micturition in about 40%, recurrent UTIs in 73%, abdominal pain 22%, and dysuria 15% [4]. The presence of fecaluria in an RUF is known to be a poor prognostic sign, indicating that the fistula may be significant in size [1, 6, 10].

The diagnosis of PF is based on history, physical examination, and imaging tests. Radiologic evaluation (CT scan and MRI) and diagnostic procedures (voiding cystourethrogram, retrograde urethrography, cystoscopy, colonoscopy) help us to delineate anatomy and identify concomitant colorectal, urethral, or bladder pathology. Urinary and fecal incontinence and function should be evaluated since they may affect subsequent treatment decisions. In our cohort, the combination of cystoscopy, retrograde urethrogram, and MRI serves as the gold standard imaging modality for diagnosing PF.

The conservative approaches used in our cohort included SPC, TUC, or PRP injection. Two (15.4%) patients observed spontaneous healing after conservative treatment with TUC or SPC. Although PRP injections failed to close the fistula, we observed a significant decrease in its size at the time of surgery compared to initial MRI data. Despite the majority of conservative management approaches failing, we believe that PRP injections or other healing-promoting factors may have a potential role. However, more data from larger trials are required to establish the efficacy of these agents in the management of these rare fistulas. Previous studies have demonstrated that conservative management of RUFs has a wide range of success rates, from 14 up to 100%, and it includes surgical procedures aimed to accomplish a urinary diversion (SPC, nephrostomy) or a fecal diversion (ileostomy or colostomy) [4]. If the RUF after prostatectomy is not closed after 3 months of catheterization, further treatment should be considered [3].

This study shows a cumulative fistula closure rate using conservative or surgical treatment of 92.3%. A meta-analysis on RUF, including 416 patients in 26 studies, showed an overall healing rate of 88%, with an overall permanent fecal or urinary diversion rate slightly higher than 20% [6]. In the case of simple classic RUF, a 3-month waiting period before surgery to give time for the lesion to spontaneously close is recommended [4]. In the complex RUFs, the interval should be increased up to 6 months to improve tissue quality. In fecal diversion, these should be closed during the first 3 months.

One crucial point regarding the surgical repair of RUFs is to distinguish and classify RUFs into simple and complex. All of the following are characteristics of RUF complexity: size larger than 2 cm, urethral stricture, bladder neck necrosis, or ischemic damage associated with ablative energies. On the other hand, a fistula is considered simple when it is secondary to surgical trauma on a previous healthy tissue [4]. In two of our patients, following the failure of conservative therapy with PRP injection and unsuccessful repair solely with prostatectomy, a second intervention for PF repair was necessary. Based on the classification of RUFs, these were categorized as complex PFs resulting from preoperative radiation for CRC.

Similar to the management of complex vesicovaginal fistulas after radiotherapy, secondary revision surgery is sometimes necessary to achieve closure [11]. We should counsel our patients that although this complication is formidable, there is a chance for cure, especially in tertiary centers specialized in reconstructive pelvic surgery. Initially, most cases required definitive urinary or fecal diversion. However, as our management strategies evolved and became more nuanced, we have achieved cure rates exceeding 80%, even in complex cases involving prior radiation therapy.

There are up to 40 different surgical approaches described to treat RUFs, including open abdominal as well as laparoscopic and robot-assisted surgery, trans-perineal, trans-anal (Parks, Latzko), trans-sphincteric (York-Mason), trans-sacral, and combined approaches [2, 4, 6]. While the principles of fistula excision remain consistent, repairing iatrogenic PF represents a unique challenge due to the involvement of the rectal cavity, prostatic gland, and prostatic urethra. Any closure attempt should consider prostate removal, which can be particularly challenging in cases involving PCa, previous radiotherapy for rectal/anal cancer, or other cancer-related treatments that may alter the normal pelvic anatomy. The transabdominal approach allows primary fistula repair or en bloc fistula removal via cysto-/prostatectomy or anterior exenteration. In experienced hands, the trans-perineal approach has some of the highest closure rates, at 91%. However, these cure rates have been reported following prostatectomy [6]. Alternatively, a trans-sphincteric approach can be considered, but generally only in non-irradiated patients, with closure rates of 85–88%, and it is usually used in non-radiated patients. In our cohort, due to the increased number of patients with various procedures for rectal/anal cancer, these procedures were considered oncologically unsafe.

In complex RUFs, the use of interposition flaps is recommended. The different flaps used are gracilis muscle, omentum, rectus muscle, gluteus maximus muscle, and dartos, with the most commonly used muscle being the gracilis (95% of cases). The gracilis flap can be an ideal tissue transfer candidate as it provides robust, well-vascularized tissue that can be interposed at the target side [4, 6]. In our cohort, flaps were used in only 4 (31%) cases, but considering the high cure rate, we do not consider this a limitation of our study. However, we recommend the usage of flaps in all cases with uro-enteric fistulas if it’s technically feasible.

### Limitations

Despite reporting the first series of acquired PF, this study was limited by its retrospective nature, the heterogeneity of the study population, and the limited number of patients. Patient follow-up was variable among investigators. Given the low rate of prostatic fistulas and the non-uniformity of these cases, a prospective analysis of operative management was not feasible. Also, an evaluation of urinary continence following fistula repair was not performed due to the heterogeneity of the study group. The majority of patients presented a temporary urinary diversion before surgery, and a preoperative evaluation of the urinary continence could not have been performed in a standardized fashion using questionnaires.

## Conclusion

Prostatic fistula is a rare but severe complication after an alternative approach in focal ablation for prostate cancer, surgery for colorectal cancer, or pelvis radiation therapy. Clinical suspicion and detailed diagnosis before surgery are essential for adapting and successfully implementing an appropriate treatment plan. Prostatic fistulas remain a crucial challenge, particularly after radiotherapy, which requires more complex reconstructive surgery. Our data have indicated the failure of conservative therapy in most of the patients with prostatic fistulas. Acquired PFs have a higher closure rate than acquired RUFs. In complex fistula, a multidisciplinary approach is mandatory, and successful PF closure can be achieved through individualized therapy based on etiology, severity, and recurrence status of PF.
